# Oral herbal medicine for women with intrahepatic cholestasis in pregnancy: a systematic review of randomized controlled trials

**DOI:** 10.1186/s12906-020-03097-x

**Published:** 2020-10-07

**Authors:** Ruiting Wang, Nuo Cheng, Rongyan Peng, Zeyu Yu, Mengdie Nan, Huijuan Cao

**Affiliations:** 1 Dongzhimen Hospital, Beijing University of Chinese Medicine, Beijing, China; 2 Beijing University of Chinese Medicine, Beijing, China; 3 Centre for Evidence Based Chinese Medicine, Beijing University of Chinese Medicine, Beijing, China

**Keywords:** Herbal medicine, Intrahepatic cholestasis, Meta-analysis, Pregnancy, Systematic review

## Abstract

**Background:**

Intrahepatic cholestasis of pregnancy (ICP) is a pregnancy complication whose range has been calculated to be between 0.01 and 15.6% all around the world. We wanted to systematically evaluate the effect and safety of oral herbal medicine on treatment for ICP.

**Methods:**

Details of the methods could be found in the registered protocol on PROSPERO (CRD42018096013). Trials assessing the effectiveness of herbal medicine for ICP were searched from seven electronic databases from inception to 28th February 2020. RevMan 5.3 software was used to perform all statistical analysis. Meta-analysis, additional analysis, Trial Sequential Analysis (TSA) and Grading of Recommendations Assessment, Development and Evaluation (GRADE) were conducted if data permitted.

**Results:**

Totally 43 randomized controlled trials with 3556 patients were included. Meta-analysis showed potential good adjunctive effect of herbal medicine on decreasing the pruritus scores (MD -0.58, 95% CI − 0.79 to − 0.36), the serum TBA scores (MD − 3.99 μmol/L, 95% CI − 4.24 to − 3.74) on the basis with Ursodesoxycholic acid. Compared to the medicine alone, significantly lower incidence of fetal distress (RR 0.41, 95% CI 0.32 to 0.51), asphyxia neonatorum (RR 0.35, 95%CI 0.25 to 0.49), cesarean section (RR 0.73, 95% CI 0.63 to 0.85), postpartum hemorrhage (RR 0.45, 95% CI 0.28 to 0.72) were observed in the combination group. But the comparison between herbal medicine and medicine showed inconsistent results among trials. Insufficient information could be used to evaluate the safety of herbal medicine for ICP.

**Conclusion:**

This review found the current evidence may support the effectiveness of combination of herbal medicine and conventional medicine for decreasing the maternal pruritus scores, the serum TBA, and the number of fetal distress, or asphyxia neonatorum events related to this condition (which was supported by TSA results). Since there were obvious statistical and clinical heterogeneity among trials, and the methodological quality of the included studies was poor, the level of the evidence could only be defined as “very low” according to the GRADE criteria. Further high quality studies are still needed to testify the effectiveness and safety of herbal medicine for ICP.

## Background

Intrahepatic cholestasis of pregnancy (ICP) is a pregnancy complication with prevalence ranging between 0.01 and 15.6% [1–4]. ICP presents as persistent pruritus, typically on the skin of palms and soles, with elevated bile acid levels, and resolves with delivery [5]. Generally, the onset of ICP occurs in the second and third trimesters and disappears spontaneously after delivery, usually within weeks [5–7]. The clinical importance of ICP lies in the potential fetal risks, including spontaneous preterm birth, iatrogenic preterm birth and fetal death [8]. While the pathophysiology of ICP is still poorly understood, gene, hormone, and environment play roles. Bile acids need to enter hepatocytes or bile ducts through the action of some transporters which genetic mutations can cause cholestasis [9, 10]. Estrogen can decrease the expression of ABCB11 / BSEP gene to inhibit the function of bile salt delivery pump, or decrease the activity of Na+ / K+ ATPase to inhibit the uptake of bile acid by hepatocytes that eventually leads to intrahepatic cholestasis. Sulfated progesterone metabolites can also have an adverse effect on bile acid excretion by inhibiting the function of BSEP [11]. Although the mechanism is unknown, studies have shown that the plasma and serum selenium concentrations and glutathione peroxidase activities in ICP patients are lower than those in healthy pregnant women, and the incidence rate is higher in winter. Some environmental factors such as pesticide pollutants, erucic acid in rape and selenium deficiency in food may lead to ICP [12]. Most often the disease affects women over the age of 35 years, [13] with personal history of cholestasis associated with the use of oral contraceptives, personal or family history of cholestasis of pregnancy, [14] biliary disease, [1] or liver disease, in multiple gestation pregnancy, [15] or in vitro fertilisation pregnancies [16]. Likewise, seasonal variations, [17] low selenium intake, erucic acid, increased gut absorption of bacterial endotoxins, pollutants, infections, and medicine are factors suspected as causing the disease [15–20].

When treating the ICP, doctors always focused on reducing maternal symptoms, improving results of liver tests, and reducing total bile acid (TBA) concentration. They commonly used Ursodeoxycholic acid (UDCA), S-adenosylmethionine (SAMe), dexamethasone, or cholestyramine as well as vitamin K (preventing postpartum bleeding) as therapies [20]. Whlie there was insufficient evidence to recommend early-term delivery or to support therapies above according to one Cochrane review [21]. However, the review found that UDCA seemed to improve the maternal symptom of pruritus, which agreed with the result of a meta-analysis by Bacq [22]. Bacq strongly suggested that UDCA was also beneficial for the fetal outcome, including total prematurity, fetal distress and neonatal respiratory distress syndrome; however, the Cochrane Review did not agree [21].

Some herbal medicine, such as Emodin, [23–25] Bushen Granule and Bushen Rougan Recipe, which includes Biejia (shell of *Trionyx sinensis* Wiegmann), Ejiao (made from skins of *Equus asinus* L.), Dihuang (tuberous root of *Rehmannia glutinosa* Libosch.), Gouqi (fruit of *Lycium barbarum* L.), Beishashen (root of *Glehnia littoralis* Fr. Schmidtex Miq.), Maidong (tuberous root of *Ophio pogon japonicas* (L. f) KerGawl.), Danggui (root of *Angelica sinensis* (Oliv.) Diels), Taoren (seed of *Prunus persica* (L.) Batsch), Qiancao (root of *Rubia cordifolia* L.), Baishao (root of *Paeonia lactiflora* Pall.), Huangjing (tuberous root of *Polygonatum kingianum* Coll. et Hemsl.), Jineijin (gizzard endothelium of *Gallus Gallus domesticus*), Dilong (*Pheretima asperigillum*), Haipiaoxiao (inner shell of *Sepiella maindroni de Rochebrune*), [26, 27] and in vivo cultured Calculus Bovis (made from bile of *Bos taurus domesticus* Gmelin) [28] were proved to be protective on cholestatic hepatitis by decreasing the levels of alanine aminotransferase (ALT) and total bile acid (TBA), prevent toxic compounds overaccumulation in hepatocytes. Decoctions like Zhi-Zi-Da-Huang decoction (including Zhizi (fruit of *Gardenia jasminoides* Ellis), Dahuang (*Rheum palmatum* L.), Zhishi (fruit of *Citrus aurantium* L.), Dandouchi (made from seeds of *Glycine max* (L.) Merr.) [29] and Shuangcao Tuihuang Granule-1 (including Yinchen (*Artemisia scoparia* Waldst. et Kit. Or *Artemisia capillaries* Thunb.), Huhuanglian (tuberous root of *Picrorhiza scrophulariiflora* Pennell), Tuxiangru (*Origanum vulgare* L.), Cheqianzi *(Plantago asiatica* L. or *Plantago depressa* Willd.), Shengdihuang (tuberous root of *Rehmannia glutinosa* Libosch.), Houpo (bark of *Magnolia officinalis* Rehd. et Wils.), etc.) [30] may also significantly dose-dependently reduce the indices of liver injuries by raising Superoxide Dismutase activity, scavenging oxygen free radicals and increasing anti-oxidation [28–30].

With the possible underlying mechanism of herbal medicinal for this condition, we conducted this systematic review to explore the potential effectiveness and safety of oral administration of herbal medicine in treating ICP.

## Methods

### Protocol registration

Protocol of this review was registered in PROSPERO as Ruiting Wang, Rongyan Peng, Nuo Cheng, Zeyu Yu, Mengdie Nan, Huijuan Cao. Oral herbal medicine for women with intrahepatic cholestasis in pregnancy: a systematic review of randomized controlled trial. PROSPERO 2018 CRD42018096013. Available from: http://www.crd.york.ac.uk/PROSPERO/display_record.php?ID=CRD42018096013.

### Eligibility criteria

Published and unpublished studies, inany language, were included where thefollowing PICOS (patient, intervention, comparator, outcome, study type) criteria were met:

i) Patient: Patients, of any age, with intrahepatic cholestasis in pregnancy. Intrahepatic cholestasis in pregnancy (ICP or OC), diagnosed in accordance with recognized criteria (e.g. Guidelines for diagnosis and treatment of intrahepatic cholestasis in pregnancy (2015) by Chinese Medical Association). ii) Intervention: Herbal medicine through oral administration. iii) Comparators: Controls include placebo and conventional medicine (such as UDCA, SAMe, dexamethasone, vitamin K etc.). Chinese medicine combined with conventional medicine compared to the conventional medicine alone are also included. The controls in protocol were selected as Guidelines for diagnosis and treatment of intrahepatic cholestasis in pregnancy (2015) by Chinese Medical Association. After analyzing the data, most of the controls were not reported in the study, so there are some differences. iv) Outcomes: Primary outcomes included the changes in Ribalta score, maternal serum TBA values, and incidence of adverse birth events (e.g. newborn deaths, fetal distress and suffocation). Secondary outcomes included changes in bile acid content, incidence of cesarean sections, postpartum hemorrhage, adverse effects of medicine, changes in ALT values, and changes in AST values for the maternal, as well as the averages gestational age at birth and the incidence of premature births. v) Study type: Randomized controlled trials.

Literatures that unable to obtain the analysable data, as well as the piratical documents would be excluded.

### Searching strategy

We searched seven databases and two clinical trial registration systems, including PubMed, SpringerLink, ProQuest, the Cochrane Central Register of Controlled Trials (CENTRAL), the Chinese National Knowledge Infrastructure Databases (CNKI), the Chongqing VIP Chinese Science and Technology Periodical Database (VIP), Wanfang Data Knowledge Service Platform, Chinese Clinical Trial Registry (ChiCTR), and Clinical Trials (ClinicalTrials.gov). The subject of the retrieval is: “Drugs, Chinese Herbal” [Mesh] OR “Herbal Medicine” [Mesh] OR “Herbal” [Mesh] with “Pregnancy” [Mesh] OR “Pregnancy Complications” [Mesh] OR “Infant” [Mesh] OR “Infant, Newborn” [Mesh] OR “Fetus” [Mesh] OR “Fetal Development” [Mesh] OR “Prenatal Diagnosis” [Mesh] OR “Fetal Monitoring” [Mesh] OR “Fetal Therapies” [Mesh] OR “Extraembryonic Membranes” [Mesh] OR “Placenta” [Mesh] OR “Placental Function Tests” [Mesh] OR “Uterine Monitoring” [Mesh] OR “Pelvimetry” [Mesh] OR “Oxytocics” [Mesh] OR “Tocolytic Agents” [Mesh] OR “Tocolysis” [Mesh] OR “Maternal Health Services” [Mesh] OR “Peripartum Period” [Mesh] OR “Parity” [Mesh] OR “Perinatal Care” [Mesh] OR “Postpartum Period” [Mesh] OR “Labor Pain” [Mesh] OR “Obstetrical” [Mesh] OR “Maternal-Child Nursing” [Mesh] OR “Midwifery” [Mesh] OR “Apgar Score” combined with “Cholestasis, Intrahepatic” [Mesh] AND “Random*” to be adjusted for use in the different databases.

Meantime, the Chinese National Knowledge Infrastructure Databases (CNKI), Wanfang Data Knowledge Service Platform and ProQuest Dissertations were used to search for grey literature.

### Data extraction (selection and coding)

Trials retrieved using the search strategy and those from additional sources were screened independently by two review authors to identify trials that potentially meet the inclusion criteria outlined above. The full texts of these trials were retrieved and independently assessed for eligibility by other two reviewers. Any disagreement was resolved through discussion with a third reviewer (Huijuan Cao). A predesigned form was used to extract data from the included trials for assessment of trial quality.

Extracted information included: i) General information: Including document number, title, first author, year(s) conducted, location (city, country), source, etc.; ii) Methodological related information: type of design, grouping number, random allocation method, Random concealment method, method of blinding, participants blinded, loss of follow up, report of selective outcome, calculation of sample size, baseline comparability; iii) Participants information: diagnostic criteria, inclusion criteria, exclusion criteria, source, sample size, age, gender, disease course. iv) Intervention information: Types of interventions, intervention performer, treatment duration; v) Outcome measures: Treatment outcomes: changes in Ribalta score, changes in TBA values, number of neonatal deaths, number of fetal distress or asphyxiation, changes in ALT values, changes in AST values, number of caesarean sections, postpartum hemorrhage, adverse medicine effects, average gestational age at birth, number of premature deliveries.

### Risk of bias assessment

We assessed the methodological quality of the included trials using the risk of bias tool recommended by the Cochrane Collaboration (Higgins and Green, 2009). Seven elements were assessed: random sequence generation, allocation concealment, blinding of participants and personnel, blinding of outcome assessment, incomplete outcome data (according to record the missing data and the method to deal with it), selective reporting (determined by the consistency of the predefined and reported outcomes) and other bias (assessed according to sample size calculation, inclusion/exclusion criteria for participant recruitment, comparability of baseline data, funding sources).

### Strategy for data analysis

All statistical analyses were performed using RevMan 5.3 (The Cochrane Collaboration) software. The results of each single trial were described. We presented results as risk ratio (RR) with its 95% confidence intervals (CI) for dichotomous outcomes, and mean difference (MD) with 95% CI for continuous outcomes. Statistical heterogeneity between the included trials were assessed using the *I*^*2*^ value, and a meta-analysis were conducted if it had proved to be no significant clinical (relating to the participants, interventions, controls, and outcomes) and statistical heterogeneity (*I*^*2*^ values are less than 75%) among the included trials. If the *I*^*2*^value was less than 25%, a fixed-effect model (FEM) was used to synthesize the data, and if the *I*^*2*^ value was between 25 and 75%, we explored the sources of the heterogeneity. If the results of sensitive analysis or subgroup analysis explained the statistical heterogeneity successfully (i.e., *I*^*2*^ value is less than 25%), we used the FEM to synthesize the data as well, otherwise, random effect model (REM) was used to combine the data. Data would not be synthesized if there was a significant level of statistical heterogeneity (i.e., *I*^*2*^ value is greater than 75%) which was could not be explain or to handle (by subgroup analysis).

### Additional analysis

If one meta-analysis included more than 10 trials, we explored the possibility of publication bias by using a funnel plot.

If one meta-analysis contained more than 8 included studies, we performed a Trial Sequential Analysis (TSA) was performed if there were more than 8 included studies in the meta-analysis. We calculated the required sample size of each meta-analysis and tested the robustness of the result with the TSA versioned 0.9.5.10 (Copenhagen: The Copenhagen Trial Unit, Center for Clinical Intervention Research, 2017). The information size required by the diversity-adjustment that we used was estimated from a control event proportion of the included trials and a priori intervention effect of 5%, and the diversity we used was estimated in the included trials.

### Evidence quality assessment

The Grading of Recommendations Assessment, Development and Evaluation criteria (GRADE) was conducted to assess the certainty of the evidence for each primary outcomes with meta-analysis. Considering the following aspects, such as study design, risk of bias, outcome consistency of trials, directness and accuracy of evidence and possibility of publication bias, we judged whether to downgrade the evidence. Finally, the evidence was rated at four levels: high, moderate, low or very low.

## Results

### Selection results

**Fig. 1:**
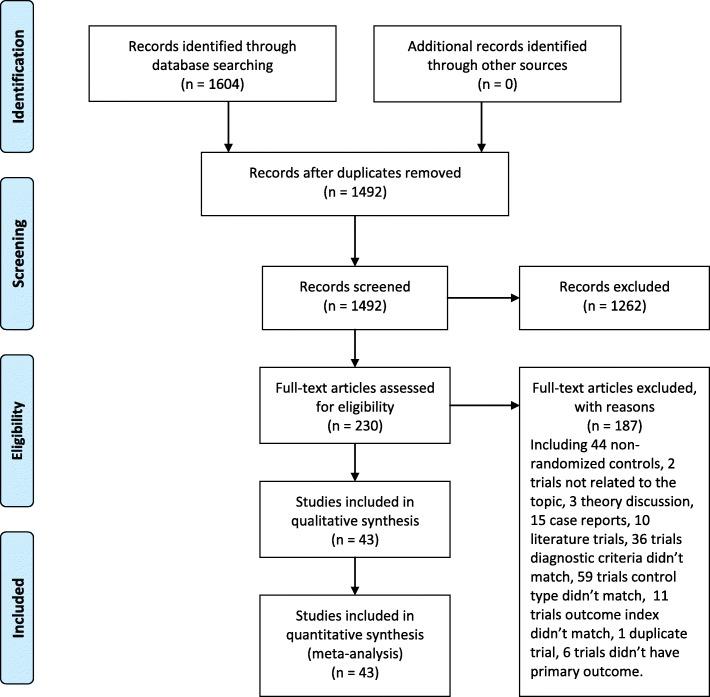
Study Flow Chart

A total of 1604 trials were retrieved according to the search strategy, and the literature was screened according to the predefined criteria. After reading the title and abstract, 1262 trials were screened out, and further another 187 trials were excluded after reading the full text. Forty-three randomized controlled trials were finally included. All the included trials were published in Chinese. The specific literature screening process is shown in Fig. 1.

### Basic characteristics of trials (see Table 1)

A total of 3556 women with ICP were included in the 43 trials [31–73]. The sample size ranged from 30 to 188 (an average of 41 patients in each group). The age distribution ranged from 18 to 42 years old, and the range of gestational age was 18–40 weeks. All cases were recruited from the outpatient/inpatient Department of Obstetrics and Gynaecology. Thirty-eight trials reported baseline comparisons in terms of demographic characteristics, and the other five trials only mentioned randomization, which did not explain whether the baseline characteristic of the participants was comparable.

**Table 1:** Characteristics of the 43 included studies

Study ID	Sample size (T/C)	Age (yrs, MD ± SD)	Gestational age of onset (wks, MD ± SD)	Gestational times	Intervention	Control	Course of treatment	Outcome
**Comparison 1. Chinese medicine vs. Conventional medicine**								
Zhang [31], 2006	48/50	26.7	28–38	Primipara	Self prescribed prescription	SAMe+Reduced glutathione	7d a course of treatment, a total of 2 courses	4, 6, 11
Yang [32], 2015	30/30	T:27.2 ± 4.0 C:27.4 ± 3.9	Not reported	M/P:11/19 M/P:13/17	Yinzhihuang oral liquid	UDCA	21d	2, 5, 9, 12
Li et al. [33], 2015	60/60	T:30.5 C:28.8	T:32.4 C:33.4	T:1.44 C:1.54	Yinchenhao Decoction	UDCA+SAMe	30d for Chinese medicine,10 d for medicine	2, 6, 9, 10, 11
Huang and Liu [34], 2004	35/25	T:34.5 ± 1.9 C:35.1 ± 2.2	T:34.5 ± 1.9 C:35.1 ± 2.2	Primipara	Yinchenhao Decoction	SAMe	21d	1, 3, 4, 6, 9, 11
Zhang et al. [35], 2006	31/29	26.8	29–40	Not reported	Jiaweiyinchen Decoction	DXM	7d	1, 3, 4, 6, 7, 9, 10, 12
**Comparison 2. Chinese medicine + Conventional medicine vs. Conventional medicine**								
Wang et al. [36], 2016	40/40	T:29 ± 4.1 C:29.5 ± 4.2	Not reported	Not reported	Yinzhihuang oral liquid+C	UDCA+SAMe	7d	2, 4, 6, 9, 10, 12
Zhou et al. [37], 2017	30/30	T:26.3 ± 2.4 C:27.6 ± 2.1	Not reported	Not reported	Yinchailishi Decoction+C	UDCA+SAMe	14d	1, 2, 4, 5, 6, 12
Liu et al. [38], 2015	78/78	T:26.5 ± 7.4 C:26.2 ± 7.6	Not reported	T:1.4 ± 0.8 C:1.5 ± 0.7	Yinchenlidan Decoction+C	SAMe+DXM	14d	1, 2, 4, 6, 9, 10
Wang X et al. [39], 2016	55/55	T:25.1 ± 4.2 C:25.3 ± 3.8	Not reported	Not reported	Danyu Decoction+C	UDCA	14d	2, 5, 6, 7, 9, 12
Yu [40], 2017	29/28	T:30.4 ± 3.5 C:31.2 ± 3.6	Not reported	Not reported	Yinzhijiangdansuan Decoction+C	UDCA(+DXM)	7d a course of treatment, a total of 2 courses	2, 4, 5, 6, 7, 12
Zhao [41], 2011	60/60	21–38	21–38	Not reported	Qingyulidan Decoction+C	DXM + SAMe+phenobarbital	7d	1, 2, 4, 5, 6, 7, 9, 10, 12
Wang and Lai [42], 2011	35/35	T:24.3 ± 4.5 C:25.3 ± 3.5	T:37.0 ± 2.1 C:36.4 ± 2.6	Not reported	Yinchenlidan Decoction+C	UDCA	10d	1, 9
Shen and Tao [43], 2009	30/30	T:25.3 ± 5.3 C:25.8 ± 4.9	T:37.3 ± 3.3 C:36.0 ± 3.6	Not reported	Tuihuangguyuan Decoction+C	SAMe	10d	1, 4, 5, 6, 7, 9, 12
Wen et al. [44], 2014	60/60	T:26.3 ± 2.4 C:26.8 ± 2.2	T:33.2 ± 3.1 C:33.5 ± 3.4	Not reported	Self prescribed prescription+C	SAMe	14d	1, 12
Lu [45], 2013	35/24	T:26.3 ± 2.4 C:25.0 ± 2.9	Not reported	Not reported	Yinchenzhuyedihuang Decoction+C	UDCA	12d	2, 4, 5, 6, 9, 12
Liu et al. [46], 2013	15/15	26.45013	Not reported	Not reported	Yinchendanshao Decoction+C	SAMe	10d	1, 2, 9, 10
Tian et al. [47], 2016	39/39	T:25.5 ± 6.2 C:25.7 ± 5.8	Not reported	Not reported	Kangdanyu Decoction+C	Reduced glutathione+UDCA+SAMe+DXM	14d	4, 5, 6, 7, 12
Ding et al. [48], 2010	94/94	T:26.0 ± 3.8 C:25.5 ± 2.8	Not reported	Not reported	Qingganhuayu Decoction+C	SAMe	14d	1, 5, 6, 12
Zhang et al. [49], 2014	30/30	T:25.3 ± 2.1 C:26.6 ± 3.0	Not reported	1.44 ± 0.62 1.65 ± 0.81	Qingdanzhiyang Decoction+C	SAMe+phenobarbital+DXM	14d	4, 5
Shan et al. [50], 2016	48/48	T:26.3 ± 4.7 C:24.9 ± 4.3	Not reported	Not reported	Huashilidan Decoction+C	UDCA+SAMe+DXM	20d	2, 4, 5, 7, 9, 12
Li et al. [33], 2015	60/60	T:28.40151 C:28.80151	T:34.4 ± 4.4 C:33.4 ± 4.4	T:1.44 ± 4. C:1.54 ± 4.	Yinchenhao Decoction+C	UDCA+SAMe	30d for Chinese medicine,10 d for medicine	2, 6, 9, 10, 11
Lan et al. [51], 2016	40/40	Not reported	Not reported	Not reported	Yinchenhao Decoction+C	UDCA+SAMe	7d a course of treatment, a total of 2 courses	1, 2, 4, 5, 12
Zhang [52], 2015	40/40	T:26.7 ± 2.1 C:27.0 ± 1.8	Not reported	Not reported	Yinchenhao Decoction+C	SAMe	7d a course of treatment, a total of 2 courses	2, 5, 9, 10, 12
Yin [53], 2015	30/30	T:26.2 ± 2.9 C:25.9 ± 3.0	Not reported	Not reported	Bushenqingli Decoction+C	UDCA	7d a course of treatment, a total of 2 courses	1, 2, 3, 4, 5, 6, 7, 8, 9, 10, 12
Zhang [54], 2008	44/42	26.6 ± 2.8	28–36	Not reported	Self prescribed prescription+C	DXM + phenobarbital	10d	2, 9
Chen and Mo [55], 2005	35/35	T:28.5 ± 2.4 C:27.8 ± 2.6	Not reported	Not reported	Self prescribed prescription+C	DXM + VK3	14d	3, 6, 7, 8, 9, 10
Ma [56], 2010	35/35	T:28.5 ± 2.4 C:27.8 ± 2.6	Not reported	Not reported	Self prescribed prescription+C	UDCA	14d	5, 6, 10, 12
Zhang et al. [57], 2016	40/41	T:31.4 ± 9.4 C:32.1 ± 10.9	Not reported	M/P:9/31 M/P:8/33	Self prescribed prescription+C	UDCA+SAMe+DXM	15d	2
Shu [58], 2018	48/45	T:27.6 ± 3.4 C:27.9 ± 4.0	Not reported	M/P: 11/37 M/P:10/35	Qianyinlidan Decoction+C	UDCA	7d	2, 9, 10
Deng [59], 2015	30/30	29.5	Not reported	Not reported	Yinzhihuang oral liquid+C	UDCA	10d	1, 9
Su et al. [60], 2015	37/37	T:29.4 ± 5.1 C:28.9 ± 4.8	T:33.5 ± 4.1 C:33.1 ± 4.0	M/P: 8/29 M/P:7/30	Lidan Decoction+C	UDCA+SAMe+ Magnesium isoglycyrrhizinate	14d	2, 3, 4, 7, 9, 10, 12
Wang [61], 2014	36/36	Not reported	T:32–36 C:32–36	Not reported	Kangyudan Decoction+C	UDCA	14d	3, 5, 6, 7, 12
Wang et al. [62], 2018	30/30	T:28 ± 3.12 C:29 ± 4.0	T:34 ± 1.3 C:35 ± 1.5	T:1.57 ± 0.65 C:1.47 ± 0.56	Yinzhihuang granule+C	UDCA+SAMe+DXM	14d	4, 12
Wei [63], 2016	30/30	T:30.5 ± 5.2 C:28.5 ± 6.1	T:31.5 ± 4.3 C:32.3 ± 3.6	Not reported	Self prescribed prescription+C	UDCA	14d	2, 3, 4, 6, 9, 10, 12
Zhang [64], 2017	62/62	T:29.7 ± 3.8 C:28.6 ± 4.5	T:32–37 C:31–38	Not reported	Yinzhihuang granule+C	UDCA	7d a course of treatment, a total of 2 courses	4, 6, 12
Zheng [65], 2019	42/42	T:26.9 ± 3.2 C:27.6 ± 3.3	T:33.6 ± 2.3 C:34.2 ± 2.3	M/P:22/20 M/P:24/18	Lidan Decoction+C	UDCA+SAMe +phenobarbital	14d	2, 4, 5, 7, 9, 10, 12
Gu et al. [66], 2014	47/36	T:27.8 ± 6.2 C:28.2 ± 5.0	T:31.5 ± 2.8 C:31.1 ± 3.1	T:2.2 ± 1.9 C:2.1 ± 2.0	Yinchen Decoction+C	UDCA	20d	1, 2, 9, 10, 11
Mao and He [67], 2014	59/59	T:26.2 ± 1.5 C:25.9 ± 1.4	T:34.8 ± 0.7 T:35.2 ± 0.8	Not reported	Qingganlidan Decoction+C	UDCA+SAMe	14-21d	2, 4, 5, 6, 9, 10, 12
Mao [68], 2016	39/38	T:28.3 ± 2.2 C:28.6 ± 1.9	T:29.9 ± 1.8 C:29.6 ± 2.1	Not reported	Yinchenhao Decoction+C	UDCA	7d	4, 7, 8, 12
Zhu and Huang [69], 2008	35/25	T:25.1 ± 2.8 C:23.9 ± 2.3	T:34.5 ± 1.9 C:35.1 ± 2.2	Not reported	Yinchenhao Decoction+C	UDCA	21d	3, 4, 6, 9, 11
Liu et al. [70], 2019	60/60	T:25.2 ± 2.1 C:25.4 ± 2.3	T:28.9 ± 2.1 C:29.0 ± 2.2	Not reported	Wuling pill+C	SAMe	14d	1, 4, 6, 7, 8, 9, 10, 12
Wang et al. [71], 2015	20/20	T:27.5 ± 5.1 C:26.7 ± 4.1	T:30.8 ± 1.7 C:31.1 ± 1.8	Not reported	Yinchenhao Decoction+C	SAMe	21d	1, 2, 3, 4, 5, 9, 10, 12
Du and Li [72], 2014	26/26	T:22–37 C:24–36	T:27–37 C:27–38	M/P: 3/23 M/P:4/22	Yiguanjian Decoction+C	UDCA+DXM	7d a course of treatment	4, 6, 7, 12
Zhang [73], 2005	40/40	T:25.5 ± 2.5 C:24.6 ± 3.1	T:33.8 ± 2 C:34.0 ± 2.2	Not reported	Dangguidihuang Decoction+C	SAMe	7d a course of treatment, a total of 3 courses	1, 2, 4, 6, 9

*M* multipara, *P* Primipara, *T* Treatment, *C* Control, *MD* Mean Difference, *SD* Standard Deviation, *UDCA* Ursodesoxycholic acid, *SAMe* S-adenosylmethionine, *DXM* Dexamethasone, *VK3* Vitamin K3

Outcome:

1 itching score

2 serum total bile acid

3 neonatal deaths

4 the number of fetal distress

5 the number of asphyxia neonatorum

6 number of caesarean section

7 the number of postpartum hemorrhage

8 the number of adverse drug reactions

9 serum alanine aminotransferase

10 serum aspartate aminotransferase

11 neonatal birth age

12 the number of premature births

All patients were diagnosed according to the Chinese recognized criteria, including those cited in the textbook of “Obstetrics” [31, 32, 36–40, 59–65], the “Chinese obstetrics and gynaecology” [31, 34, 35, 41–50, 66–69], the “Obstetrics and Gynaecology Section of the Chinese Medical Association Obstetrics and Gynaecology Branch: Guide to diagnosis and treatment of intrahepatic cholestasis of pregnancy” [33, 51–53, 70], the “Practical Obstetrics and Gynaecology Handbook” [54], the “TCM Gynaecology” [45, 60, 65], the “Guidelines for diagnosis and treatment of intrahepatic cholestasis of pregnancy (2015)” [40, 71], the “Clinical obstetrics and gynaecology” [72], and the Reyes standard [73].

Among the included trials, four trials compared Chinese herbal medicine with conventional medicine [31, 32, 34, 35], 38 trials [34–73] compared the combination of the herbal medicine and conventional medicine with conventional medicine alone, the remaining one three-arms trial [33] covered both of these two comparisons. As the intervention, 30 trials used herbal decoction with fixed prescriptions [33–35, 37–43, 45–53, 58, 60, 61, 65–69, 71–73], seven trials used self-made prescriptions according to syndrome differentiation principle [31, 44, 54–57, 63], six trials used herbal patent [32, 36, 59, 62, 64, 70]. Main herbal prescriptions (reported in two studies or more) was Yinchenhao Decoction (*n* = 7). Main herbs (reported in twenty studies or more) included: Yichen (*Artemisiacapillaris thunb*) (*n* = 38), Huangqin (*Scutellaria baicalensis* Georgi) (*n* = 29), Zhizi (*Gardenia jasminoides* Ellis) (*n* = 28), Dahuang (*Rheum officinale* Baill) (*n* = 21), Fuling (*Poria cocos* (Schw.) Wolf.) (*n* = 20). Herbal patents included Yinzhihuang oral liquid [32, 36, 59, 64], Yinzhihuang granule [62], Wuling pill [70]. The ingredients of them were showed in Supplementary material 1. Treatment durations varied from 7 to 21 days among the included trials.

Forty-three trials [31–73] used conventional medicine as control treatment, including Ursodesoxycholic acid (UDCA) [39, 42, 45, 53, 56, 58, 59, 61, 63, 64, 66, 68, 69] (P.O., 8 ~ 15 mg/kg/d, 1 ~ 3 times/d), S-adenosylmethionine (SAMe) [32, 34, 43, 44, 46, 48, 52, 70, 71, 73] (P.O., 1 ~ 2 g/d; or ivgtt., 0.8 ~ 1 g/d, 5% glucose injection 250 mL), Dexamethasone [35] (P.O., 9 mg/d, 3 times/d), Dexamethasone plus vitamin K3 [55], SAMe plus reduced glutathione (GSH) [31], UDCA plus SAMe [33, 36, 37, 51, 67], SAMe plus phenobarbital and dexamethasone [41, 49], Dexamethasone plus Phenobarbital [54], UDCA plus SAMe and dexamethasone [50, 57, 62], SAMe plus dexamethasone [38] (SAMe: ivgtt., 1.0 g/d; dexamethasone: i.m., 6 ~ 10 mg, 1 ~ 2 times/d), GSH plus UDCA and dexamethasone [47], UDCA plus SAMe plus magnesium isoglycyrrhizinate [60], UDCA plus phenobarbital plus SAMe [65], UDCA plus dexamethasone (for patients with tendency of premature birth) [40, 72].

Twenty-two trials reported the changes in the pruritus score [34–38, 41–44, 46–48, 50–53, 59, 66, 69–71, 73]. The pruritus score of 18 trials was based on the standard Ribalta pruritus score [34, 35, 37, 38, 41–44, 46, 48, 51, 53, 59, 66, 69–71, 73]. Twenty-seven trials reported the post-treatment TBA values [31–33, 36–41, 45–48, 50–55, 57, 58, 60, 63, 66, 67, 71, 73]. Among them, 24 trials reported the serum TBA values [31–33, 36–41, 45, 46, 50–55, 57, 60, 63, 66, 67, 71, 73]. The TBA detection method of the remaining three studies is unknown [47, 48, 58]. One trial reported the changes in the serum TBA values after treatment [49]. Nine trials reported the neonatal death toll [34, 35, 53, 55, 60, 61, 63, 69, 71]. Twenty-seven trials reported the number of fetal distress [31, 34–38, 40, 41, 43, 45, 47, 49–51, 53, 60, 62–65, 67–73]. Nineteen trials reported the number of asphyxia neonatorum [32, 37, 39–41, 43, 45, 47–53, 56, 61, 65, 67, 71]. Besides the above primary outcomes we defined in this review, 25 trials reported the number of cesarean section [31, 33–41, 43, 45, 47, 48, 53, 55, 56, 61, 63, 64, 67, 69, 70, 72, 73], 15 trials reported the number of postpartum hemorrhage [35, 39–41, 43, 47, 50, 53, 55, 60, 61, 65, 68, 70, 72], five trials reported the number of adverse events [48, 53, 55, 68, 70]. Twenty-nine trials reported the post-treatment serum ALT values [32–36, 38, 39, 41–43, 45, 46, 49–55, 58–60, 63, 65–67, 69–71, 73], and 19 trials reported the post-treatment serum AST values [33, 35, 36, 38, 41, 46, 49, 50, 52, 53, 55, 58, 60, 63, 65–67, 70, 71]. Five trials reported the average gestational age of the newborn [31, 33, 34, 66, 69]. Twenty-seven trials reported the number of premature birth [32, 35–37, 39–41, 43–45, 47, 48, 50–55, 60, 62–65, 67, 68, 70–72], and one trial reported the number of premature birth before gestational age of 37 weeks [45].

### Quality evaluation of inclusion studies

**Fig. 2:**
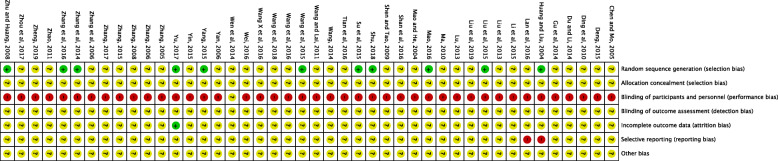
Risk of Bias assessment for the included trials

None studies used the appropriate random number table method for sequence generation [32, 34, 38, 49, 57, 60, 68, 69, 71], one study used coinage method to generate random numbers [58]. It is unclear whether the remaining studies had used adequate randomization allocation methods. None of the 43 studies reported the allocation concealment or blinding of participants or investigators. One study reported and explained the dropouts and losses to follow-up [40], and none of the others reported dropouts. Two study has selective reporting outcomes [34, 51], because one of them intended to use liver function data as outcome measures but was not reported in result and another one reported the number of adverse birth events but was not reported in protocol. All studies did not report sample size estimates and financial support, also the baseline comparability was unclear. Thus, all the included trials were considered to be at high risk of other bias. So all the other bias risks of the 43 studies are unclear. In summary, all of the included trials were assessed as having high risk of bias due to the undefined methods of randomization, absence of blinding and the potential inappropriate method on dealing with missing data (See Fig. 2).

### Estimate effects (see Table 2)

#### Herbal medicine vs. conventional medicine

A total of five studies compared herbal medicine with liver protection medicine [31–35].

**Table 2:** Effect of estimates of oral herbal medicine for intrahepatic cholestasis in pregnancy in 43 included trials

Trials	Intervention/Comparison	Effect estimates (95%CI)	***P***value
**1. The pruritus score of the pregnancy**			
1.1 Herbal medicine vs. Conventional medicine			
Huang and Liu [34], 2004	Yinchenhao Decoction	MD − 0.70 [− 0.93, − 0.47]	
Zhang et al. [35], 2006	Jiaweiyinchen Decoction	MD − 0.55 [− 1.07, − 0.03]	
**Subtotal (REM,** ***I***^**2**^ **= 0%)**		**MD − 0.68 [− 0.88, − 0.47]**	**< 0.00001**
1.2 Herbal medicine plus Conventional medicine versus Conventional medicine			
*1.2.1 Herbal medicine plus SAMe* versus *SAMe*			
Shen and Tao [43], 2009	Tuihuangguyuan Decoction	MD − 0.65 [− 1.02, − 0.28]	
Wen et al. [44], 2014	Self prescribed prescription	MD-1.27 [− 1.47, − 1.07]	
Liu et al. [46], 2013	Yinchendanshao Decoction	MD − 0.50 [− 1.16, 0.16]	
Ding et al. [48], 2010	Qingganhuayu Decoction	MD − 1.20 [− 1.57, − 0.83]	
Liu et al. [70], 2019	Wuling pill	MD − 1.02 [− 1.08, − 0.96]	
Wang et al. [71], 2015	Yinchen Decoction	MD − 0.70 [− 1.10, − 0.30]	
Zhang [73], 2005	Dangguidihuang Decoction	MD − 0.70 [− 0.86, − 0.54]	
*1.2.2 Herbal medicine plus UDCA* versus *UDCA*			
Wang and Lai [42], 2011	Yinchenlidan Decoction	MD − 0.70 [− 1.01, − 0.39]	
Yin [53], 2015	Bushenqingli Decoction	MD − 0.70 [− 1.13, − 0.27]	
Deng [59], 2015	Yinzhihuang oral liquid	MD − 0.68 [− 0.91, − 0.45]	
Gu et al. [66], 2014	Yinchen Decoction	MD −0.32 [− 0.54, − 0.10]	
**Subtotal (REM,** ***I***^**2**^ **= 56%)**		**MD − 0.58 [− 0.79, − 0.36]**	**< 0.00001**
*1.2.3 Herbal medicine plus UDCA plus SAMe* versus *UDCA plus SAMe*			
Zhou et al. [37], 2017	Yinchailishi Decoction	MD −0.77 [− 1.01, − 0.53]	
Lan et al. [51], 2016	Yinchenhao Decoction	MD − 1.68 [− 1.99, − 1.37]	
**Subtotal (REM,** ***I***^**2**^ **= 95%)**		**MD − 1.22 [− 2.11, − 0.33]**	**< 0.00001**
*1.2.4 Herbal medicine plus dexamethasone* versus *dexamethasone*			
Liu et al. [38], 2015	Yinchenlidan Decoction	MD −0.70 [− 0.88, − 0.52]	
Zhao [41], 2011	Qingyulidan Decoction	MD −0.68 [− 0.83, − 0.53]	
**Subtotal (FEM,** ***I***^**2**^ **= 0%)**		**MD − 0.69 [− 0.80, − 0.57]**	**< 0.00001**
**2. Maternal serum TBA**			
2.1 Herbal medicine vs. Conventional medicine			
Yang [32], 2015	Yinzhihuang oral liquid vs. UDCA	MD − 4.74 [− 6.57, − 2.91]	
Li et al. [14], 2015	Yinchenhao Decoction vs. UDCA+SAM	MD 1.40 [− 0.45, 3.25]	
2.2 Herbal medicine plus Conventional medicine versus Conventional medicine			
*2.2.1 Herbal medicine plus SAMe* versus *SAMe*			
Liu et al. [46], 2013	Yinchendanshao Decoction	MD −10.66 [− 19.08, − 2.24]	
Zhang [52], 2015	Yinchenhao Decoction	MD −8.20 [− 10.04, − 6.36]	
Wang et al. [71], 2015	Yinchen Decoction	MD − 6.00 [− 8.18, − 3.82]	
Zhang [73], 2005	Dangguidihuang Decoction	MD −7.20 [− 9.39, − 5.01]	
**Subtotal (FEM,** ***I***^**2**^ **= 0%)**		**MD − 7.33 [− 8.50, − 6.15]**	**< 0.00001**
*2.2.2 Herbal medicine plus UDCA versusUDCA*			
Wang X et al. [39], 2016	Danyu Decoction	MD − 6.44 [− 9.73, − 3.15]	
Yu [40], 2017	Yinzhijiangdansuan Decoction	MD −4.33 [− 7.48, − 1.18]	
Lu [45], 2013	Yinchenzhuyedihuang Decoction	MD − 3.51 [− 4.88, − 2.14]	
Yin [53], 2015	Bushenqingli Decoction	MD −4.45 [− 5.76, − 3.14]	
Shu [58], 2018	Qianyinlidan Decoction	MD −3.94 [− 4.20, − 3.68]	
Wei [63], 2016	Self prescribed prescription	MD −5.00 [− 6.78, − 3.22]	
Gu et al. [66], 2014	Yinchen Decoction	MD −4.81 [− 7.25, − 2.37]	
**Subtotal (FEM,** ***I***^**2**^ **= 0%)**		**MD-3.99 [− 4.24, − 3.74]**	**< 0.00001**
*2.2.3 Herbal medicine plus UDCA plus SAMe* versus *UDCA plus SAMe*			
Wang et al. [36], 2016	Yinzhihuang oral liquid	MD − 2.30 [− 4.18, − 0.42]	
Zhou et al. [37], 2017	Yinchailishi Decoction	MD − 7.08 [− 9.88, − 4.28]	
Liu et al. [38], 2015	Yinchenlidan Decoction	MD − 8.13 [− 9.45, − 6.81]	
Lan et al. [51], 2016	Yinchenhao Decoction	MD − 1.77 [− 4.30, 0.76]	
Su et al. [60], 2015	Lidan Decoction	MD − 3.42 [− 6.32, − 0.52]	
Mao and He [67], 2014	Qingganlidan Decoction	MD −24.10 [− 26.05, − 22.15]	
Zheng [65], 2019	Lidan Decoction	MD −6.38 [− 7.69, − 5.07]	
**Subtotal (REM,** ***I***^**2**^ **= 98%)**		**MD − 7.62 [− 12.97, − 2.27]**	**0.005**
*2.2.4 Herbal medicine plus UDCA, SAMe and dexamethasone versusUDCA, SAMe and dexamethasone*			
Zhao [41], 2011	Qingyulidan Decoction	MD-3.57 [−4.74, − 2.40]	
Shan et al. [50], 2016	Huashilidan Decoction	MD-3.54 [− 5.33, − 1.75]	
Li et al.,^14^ 2015	Yinchenhao Decoction	MD-5.00 [− 6.71, − 3.29]	
Zhang et al. [57], 2016	Self prescribed prescription	MD-3.37 [− 3.86, − 2.88]	
**Subtotal (FEM,** ***I***^**2**^ **= 8%)**		**MD-3.50 [−3.93, − 3.08]**	**< 0.00001**
**3. The number of fetal distress**			
3.1 Herbal medicine vs. Conventional medicine			
Zhang [31], 2006	Self prescribed prescription vs SAMe+Reduced glutathione	RR 0.93 [0.39,2.20]	
Huang and Liu [34], 2004	Yinchenhao Decoction vs SAMe	RR 0.86 [0.29, 2.50]	
Zhang et al. [35], 2006	Jiaweiyinchen Decoction vs DXM	RR 0.47 [0.13, 1.70]	
**Subtotal (FEM,** ***I***^**2**^ **= 0%)**		**RR 0.77 [0.43, 1.39]**	**0.39**
3.2 Herbal medicine plus Conventional medicine versus Conventional medicine			
Wang et al. [36], 2016	Yinzhihuang oral liquid plus UDCA and SAMe versus UDCA and SAMe	RR 0.22 [0.05, 0.96]	
Zhou et al. [37], 2017	Yinchailishi Decoction plus UDCA and SAMe versus UDCA and SAMe	RR 0.33 [0.01, 7.87]	
Liu et al. [38], 2015	Yinchenlidan Decoction plus UDCA and SAMe versus UDCA and SAMe	RR 0.31 [0.12, 0.81]	
Yu [40], 2017	Yinzhijiangdansuan Decoction plus UDCA versus UDCA	RR 0.39 [0.08, 1.83]	
Zhao [41], 2011	Qingyulidan Decoction plus UDCA, SAMe and dexamethasone versusUDCA, SAMe and dexamethasone	RR 0.25 [0.06, 1.13]	
Shen and Tao [43], 2009	Tuihuangguyuan Decoction plus SAMe versus SAMe	RR 0.38 [0.11, 1.28]	
Lu [45], 2013	Yinchenzhuyedihuang Decoction plus UDCA versus UDCA	RR 0.29 [0.08, 1.02]	
Tian et al. [47], 2016	Kangdanyu Decoction plus Reduced glutathione plus UDCA plus SAMe plus DXM versus Reduced glutathione plus UDCA plus SAMe	RR 1.40 [0.71, 2.76]	
Zhang et al. [49], 2014	Qingdanzhiyang Decoction plus SAMe, phenobarbital and DXM versus SAMe, phenobarbital and DXM	RR 0.27 [0.08, 0.88]	
Shan et al. [50], 2016	Huashilidan Decoction plusUDCA, SAMe and dexamethasone versusUDCA, SAMe and dexamethasone	RR 0.40 [0.08, 1.96]	
Lan et al. [51], 2016	Yinchenhao Decoction plus UDCA and SAMe versus UDCA and SAMe	RR 0.54 [0.24, 1.21]	
Yin [53], 2015	Bushenqingli Decoction plus UDCA versus UDCA	RR 0.33 [0.10, 1.11]	
Su et al. [60], 2015	Lidan Decoction plus UDCA, SAMe and Magnesium isoglycyrrhizinate versus UDCA, SAMe and Magnesium isoglycyrrhizinate	RR 0.67 [0.12, 3.76]	
Wang et al. [62], 2018	Yinzhihuang granule plus UDCA, SAMe and DXM versus UDCA, SAMe and DXM	RR 0.20 [0.05, 0.84]	
Wei [63], 2016	Self prescribed prescription plus UDCA versus UDCA	RR 0.21 [0.08, 0.55]	
Zhang [64], 2017	Lidan Decoction plus UDCA versus UDCA	RR 0.25 [0.03, 2.17]	
Zheng [65], 2019	Yinchen Decoction plus UDCA, SAMe and Phenobarbital versus UDCA, SAMe and Phenobarbital	RR 0.50 [0.05, 5.31]	
Mao and He [67], 2014	Qingganlidan Decoction plus UDCA and SAMe versus UDCA and SAMe	RR 0.31 [0.12, 0.80]	
Mao [68], 2016	Yinchenhao Decoction plus UDCA versus UDCA	RR 0.97 [0.06, 15.02]	
Zhu and Huang [69], 2008	Yinchenhao Decoction plus UDCA versus UDCA	RR 0.86 [0.29, 2.50]	
Liu et al. [70], 2019	Wuling pill plus SAMe versus SAMe	RR 0.40 [0.08, 1.98]	
Wang et al. [71], 2015	Yinchenhao Decoction plus SAMe versus SAMe	Not estimable	
Du and Li [72], 2014	Yiguanjian Decoction plus UDCA and DXM versus UDCA and DXM	RR 0.35 [0.17, 0.75]	
Zhang [73], 2005	Dangguidihuang Decoction plus SAMe versus SAMe	RR 0.50 [0.21, 1.20]	
**Overall (FEM,** ***I***^***2***^ **= 0%)**		**RR0.41 [0.32, 0.51]**	**< 0.00001**
**4. The number of asphyxia neonatorum**			
4.1 Herbal medicine vs. Conventional medicine			
Yang [32], 2015	Yinzhihuang oral liquid vs UDCA	RR 1.00 [0.06,16.76]	1.00
4.2 Herbal medicine plus Conventional medicine versus Conventional medicine			
Zhou et al. [37], 2017	Yinchailishi Decoction plus UDCA and SAMe versus UDCA and SAMe	RR 0.33 [0.01, 7.87]	
Wang X et al. [39], 2016	Danyu Decoction plus UDCA versus UDCA	RR 0.33 [0.07, 1.58]	
Yu [40], 2017	Yinzhijiangdansuan Decoction plus UDCA versus UDCA	RR 0.48 [0.05, 5.03]	
Zhao [41], 2011	Qingyulidan Decoction plus UDCA, SAMe and dexamethasone versusUDCA, SAMe and dexamethasone	RR 0.25 [0.03, 2.17]	
Shen and Tao [43], 2009	Tuihuangguyuan Decoction plus SAMe versus SAMe	RR 0.11 [0.01, 1.98]	
Lu [45], 2013	Yinchenzhuyedihuang Decoction plus UDCA versus UDCA	RR 0.29 [0.08, 1.02]	
Tian et al. [47], 2016	Kangdanyu Decoction plus Reduced glutathione, UDCA, SAMe and DXM versus Reduced glutathione, UDCA and SAMe	RR 0.85 [0.43, 1.65]	
Ding et al. [48], 2010	Qingganhuayu Decoction plus SAMe versus SAMe	RR 0.33 [0.13, 0.88]	
Zhang et al. [49], 2014	Qingdanzhiyang Decoction plus SAMe, phenobarbital and DXM versus SAMe, phenobarbital and DXM	RR 0.13 [0.02, 0.94]	
Shan et al. [50], 2016	Huashilidan Decoction plus UDCA, SAMe and dexamethasone versusUDCA, SAMe and dexamethasone	RR 0.20 [0.01, 4.06]	
Lan et al. [51], 2016	Yinchenhao Decoction plus UDCA and SAMe versus UDCA and SAMe	RR 0.50 [0.19, 1.33]	
Zhang [52], 2015	Yinchenhao Decoction plus SAMe versus SAMe	RR 0.25 [0.06, 1.11]	
Yin [53], 2015	Bushenqingli Decoction plus UDCA versus UDCA	RR 0.13 [0.02, 0.94]	
Ma [56], 2010	Self prescribed prescription plus UDCA versus UDCA	RR 0.38 [0.11, 1.30]	
Wang [61], 2014	Kangyudan Decoction plus UDCA versus UDCA	RR 0.40 [0.08, 1.93]	
Zheng [65], 2019	Yinchen Decoction plus UDCA, SAMe and Phenobarbital versus UDCA, SAMe and Phenobarbital	RR 0.33 [0.01, 7.96]	
Mao and He [67], 2014	Qingganlidan Decoction plus UDCA and SAMe versus UDCA and SAMe	RR 0.23 [0.07, 0.77]	
Wang et al. [71], 2015	Yinchenhao Decoction plus SAMe versus SAMe	Not estimable	
**Overall (FEM,** ***I***^***2***^ **= 0%)**		**RR0.35 [0.25, 0.49]**	**< 0.00001**
**5. The number of cesarean section**			
5.1 Herbal medicine vs. Conventional medicine			
Zhang [31], 2006	Self prescribed prescription vs SAMe and Reduced glutathione	RR 0.93 [0.56, 1.55]	
Li et al. [14], 2015	Yinchenhao Decoction vs. UDCA and SAM	RR 1.07 [0.85, 1.37]	
Huang and Liu [34], 2004	Yinchenhao Decoction vs SAMe	RR 0.93 [0.56, 1.55]	
Zhang et al. [35], 2006	Jiaweiyinchen Decoction vs DXM	RR 0.58 [0.22, 1.58]	
**Overall (FEM,** ***I***^***2***^ **= 0%)**		**RR 0.97 [0.80,1.18]**	**0.78**
5.2 Herbal medicine plus Conventional medicine versus Conventional medicine			
Wang et al. [36], 2016	Yinzhihuang oral liquid plus UDCA and SAMe versus UDCA and SAMe	RR 0.27 [0.08, 0.90]	
Zhou et al. [37], 2017	Yinchailishi Decoction plus UDCA and SAMe versus UDCA and SAMe	RR 0.69 [0.53, 0.90]	
Liu et al. [38], 2015	Yinchenlidan Decoction plus UDCA and SAMe versus UDCA and SAMe	RR 0.71 [0.51, 0.99]	
Wang X et al. [39], 2016	Danyu Decoction plus UDCA versus UDCA	RR 0.38 [0.23, 0.62]	
Yu [40], 2017	Yinzhijiangdansuan Decoction plus UDCA versus UDCA	RR 0.57 [0.32, 1.02]	
Zhao [41], 2011	Qingyulidan Decoction plus UDCA, SAMe and dexamethasone versus UDCA, SAMe and dexamethasone	RR 0.95 [0.73, 1.24]	
Shen and Tao [43], 2009	Tuihuangguyuan Decoction plus SAMe versus SAMe	RR 0.91 [0.67, 1.24]	
Lu [45], 2013	Yinchenzhuyedihuang Decoction plus UDCA versus UDCA	RR 0.53 [0.28, 1.00]	
Tian et al. [47], 2016	Kangdanyu Decoction plus Reduced glutathione, UDCA, SAMe and DXM versus Reduced glutathione, UDCA and SAMe	RR 0.52 [0.30, 0.89]	
Ding et al. [48], 2010	Qingganhuayu Decoction plus SAMe versus SAMe	RR 1.03 [0.89, 1.19]	
Li et al. [14], 2015	Yinchenhao Decoction plus UDCA andSAMe versus UDCA and SAMe	RR 0.80 [0.59, 1.08]	
Yin [53], 2015	Bushenqingli Decoction plus UDCA versus UDCA	RR 0.50 [0.14, 1.82]	
Chen and Mo [55], 2005	Self prescribed prescription plus DXM and VK3 versus DXM and VK3	RR 1.08 [0.83, 1.39]	
Ma [56], 2010	Self prescribed prescription plus UDCA versus UDCA	RR 1.11 [0.72, 1.71]	
Wang [61], 2014	Kangyudan Decoction plus UDCA versus UDCA	RR 0.67 [0.46, 0.97]	
Wei [63], 2016	Self prescribed prescription plus UDCA versus UDCA	RR 0.50 [0.19, 1.29]	
Zhang [64], 2017	Lidan Decoction plus UDCA versus UDCA	RR 0.27 [0.08, 0.93]	
Mao and He [67], 2014	Qingganlidan Decoction plus UDCA and SAMe versus UDCA and SAMe	RR 0.80 [0.57, 1.13]	
Zhu and Huang [69], 2008	Yinchenhao Decoction plus UDCA versus UDCA	RR 0.93 [0.56, 1.55]	
Liu et al. [70], 2019	Wuling pill plus SAMe versus SAMe	RR 0.17 [0.02, 1.34]	
Du and Li [72], 2014	Yiguanjian Decoction plus UDCA and DXM versus UDCA and DXM	RR 0.39 [0.20, 0.77]	
Zhang [73], 2005	Dangguidihuang Decoction plus SAMe versus SAMe	RR 0.95 [0.61, 1.49]	
**Overall (REM,** ***I***^***2***^ **= 65%)**		**RR 0.73 [0.63, 0.85]**	**< 0.0001**
**6. The number of postpartum haemorrhage**			
6.1 Herbal medicine plus versus Conventional medicine			
Zhang et al. [35], 2006	Jiaweiyinchen Decoction vs DXM	RR 0.47 [0.09, 2.36]	0.36
6.2 Herbal medicine plus Conventional medicine versus Conventional medicine			
Wang X et al. [39], 2016	Danyu Decoction plus UDCA versus UDCA	RR 0.50 [0.05, 5.36]	
Yu [40], 2017	Yinzhijiangdansuan Decoction plus UDCA versus UDCA	RR 0.48 [0.05, 5.03]	
Zhao [41], 2011	Qingyulidan Decoction plus UDCA, SAMe and dexamethasone versus UDCA, SAMe and dexamethasone	RR 2.00 [0.19, 21.47]	
Shen and Tao [43], 2009	Tuihuangguyuan Decoction plus SAMe versus SAMe	RR 2.00 [0.19, 20.90]	
Tian et al. [47], 2016	Kangdanyu Decoction plus Reduced glutathione, UDCA, SAMe and DXM versus Reduced glutathione, UDCA and SAMe	RR 0.25 [0.06, 1.10]	
Shan et al. [50], 2016	Huashilidan Decoction plus UDCA, SAMe and dexamethasone versusUDCA, SAMe and dexamethasone	RR 0.50 [0.05, 5.33]	
Yin [53], 2015	Bushenqingli Decoction plus UDCA versus UDCA	Not estimable	
Chen and Mo [55], 2005	Self prescribed prescription plus DXM and VK3 versus DXM and VK3	RR 0.67 [0.12, 3.75]	
Su et al. [60], 2015	Lidan Decoction plus UDCA, SAMe and Magnesium isoglycyrrhizinate versus UDCA, SAMe and Magnesium isoglycyrrhizinate	RR 0.50 [0.05, 5.28]	
Wang [61], 2014	Kangyudan Decoction plus UDCA versus UDCA	RR 1.00 [0.07, 15.38]	
Zheng [65], 2019	Yinchen Decoction plus UDCA, SAMe and Phenobarbital versus UDCA, SAMe and Phenobarbital	RR 0.33 [0.04, 3.08]	
Mao [68], 2016	Yinchenhao Decoction plus UDCA versus UDCA	RR 0.16 [0.02, 1.29]	
Liu et al. [70], 2019	Wuling pill plus SAMe versus SAMe	RR 0.40 [0.08, 1.98]	
Du and Li [72], 2014	Yiguanjian Decoction plus UDCA and DXM versus UDCA and DXM	RR 0.38 [0.16, 0.92]	
**Overall (FEM,** ***I***^***2***^ **= 0%)**		**RR 0.45 [0.28, 0.72]**	**0.0009**
**7. The serum ALT values**			
7.1 Herbal medicine vs. Conventional medicine			
Yang [32], 2015	Yinzhihuang oral liquid vs. UDCA	MD − 1.54 [− 2.46, − 0.62]	
Li et al. [14], 2015	Yinchenhao Decoction vs. UDCA and SAM	MD 5.70 [0.50,10.90]	
Huang and Liu [34], 2004	Yinchenhao Decoction vs SAMe	MD 3.40 [− 12.37, 19.17]	
Zhang et al. [35], 2006	Jiaweiyinchen Decoction vs DXM	MD − 18.31 [− 46.10, 9.48]	
**Subtotal (REM,** ***I***^***2***^ **= 67%)**		**MD 0.90 [− 5.10, 6.90]**	**0.77**
7.2 Herbal medicine plus Conventional medicine versus Conventional medicine			
*7.2.1 Herbal medicine plus SAMe* versus *SAMe*			
Shen and Tao [43], 2009	Tuihuangguyuan Decoction	MD − 11.50 [− 16.78, − 6.22]	
Liu et al. [46], 2013	Yinchendanshao Decoction	MD 2.97 [− 3.73, 9.67]	
Zhang [52], 2015	Yinchenhao Decoction	MD − 13.60 [− 19.20, − 8.00]	
Liu et al. [70], 2019	Wuling pill	MD − 43.86 [− 47.04, − 40.68]	
Wang et al. [71], 2015	Yinchenhao Decoction	MD − 10.20 [− 17.59, − 2.81]	
Zhang [73], 2005	Dangguidihuang Decoction plus SAMe versus SAMe	MD −14.02 [− 19.04, − 9.00]	
*7.2.2 Herbal medicine plus UDCA* versus *UDCA*			
Wang X et al. [39], 2016	Danyu Decoction	MD-26.10 [− 50.18, − 2.02]	
Wang and Lai [42], 2011	Yinchenlidan Decoction	MD − 72.00 [− 75.85, − 68.15]	
Lu [45], 2013	Yinchenzhuyedihuang Decoction	MD − 13.00 [− 17.50, − 8.50]	
Yin [53], 2015	Bushenqingli Decoction	MD − 19.07 [− 24.70, − 13.44]	
Shu [58], 2018	Qianyinlidan Decoction	MD − 16.98 [− 18.89, − 15.07]	
Deng [59], 2015	Yinzhihuang oral liquid	MD − 10.40 [− 16.37, − 4.43]	
Wei [63], 2016	Self prescribed prescription	MD − 11.67 [− 17.33, − 6.01]	
Gu et al. [66], 2014	Yinchenhao Decoction	MD − 1.37 [− 5.07, 2.33]	
Zhu and Huang [69], 2008	Yinchenhao Decoction	MD − 8.60 [− 24.25, 7.05]	
*7.2.3 Herbal medicine plus UDCA and SAMe* versus *UDCA and SAMe*			
Wang et al. [36], 2016	Danyu Decoction	MD − 14.85 [− 18.88, − 10.82]	
Li et al. [14], 2015	Yinchenhao Decoction	MD − 21.00 [− 25.60, − 16.40]	
Su et al. [60], 2015	Lidan Decoction	MD − 18.40 [− 23.56, − 13.24]	
Zheng [65], 2019	Yinchen Decoction	MD − 19.11 [− 20.98, − 17.24]	
Mao and He [67], 2014	Qingganlidan Decoction	MD − 21.10 [− 24.86, − 17.34]	
**Subtotal (REM,** ***I***^***2***^ **= 34%)**		**MD-18.94 [− 20.91, − 16.97]**	**< 0.0001**
*7.2.4 Herbal medicine plus UDCA, SAMe and dexamethasone versusUDCA, SAMe and dexamethasone*			
Shan et al. [50], 2016	Huashilidan Decoction	MD-8.69 [− 13.16, − 4.22]	0.0001
*7.2.5 Herbal medicine plus dexamethasone* versus *dexamethasone*			
Liu et al. [38], 2015	Yinchenlidan Decoction	MD-13.33 [− 17.34, − 9.32]	
Zhao [41], 2011	Qingyulidan Decoction	MD-10.13 [− 13.93, − 6.33]	
Zhang [54], 2008	Self prescribed prescription	MD-19.10 [− 23.90, − 14.30]	
Chen and Mo [55], 2005	Self prescribed prescription	MD-20.03 [− 27.91, − 12.15]	
**Subtotal (REM,** ***I***^***2***^ **= 72%)**		**MD-15.05 [− 19.59, − 10.51]**	**< 0.0001**
**8. The serum AST values**			
8.1 Herbal medicine vs. Conventional medicine			
Li et al. [14], 2015	Yinchenhao Decoction vs UDCA and SAM	MD 11.20 [7.86,14.54]	
Zhang et al. [35], 2006	Jiaweiyinchen Decoction vs DXM	MD −12.90 [− 37.51, 11.71]	
**Overall (REM,** ***I***^***2***^ **= 72%)**		**MD 2.36 [− 20.40, 25.12]**	**0.84**
8.2 Herbal medicine plus Conventional medicine versus Conventional medicine			
*8.2.1 Herbal medicine plus SAMe* versus *SAMe*			
Liu et al. [46], 2013	Yinchendanshao Decoction	MD-3.07 [− 7.69, 1.55]	
Zhang [52], 2015	Yinchenhao Decoction	MD-17.60 [− 26.96, − 8.24]	
Liu et al. [70], 2019	Wuling pill	MD − 35.30 [− 38.82, − 31.78]	
Wang et al. [71], 2015	Yinchenhao Decoction	MD − 6.10 [− 9.83, − 2.37]	
*8.2.2 Herbal medicine plus UDCA* versus *UDCA*			
Yin [53], 2015	Bushenqingli Decoction	MD − 20.23 [− 25.21, − 15.25]	
Ma [56], 2010	Self prescribed prescription	MD − 6.60 [− 15.02, 1.82]	
Shu [58], 2018	Qianyinlidan Decoction	MD − 29.83 [− 32.37, − 27.29]	
Wei [63], 2016	Self prescribed prescription	MD 8.00 [2.00, 14.00]	
Gu et al. [66], 2014	Yinchenhao Decoction	MD −1.76 [− 4.51, 0.99]	
*8.2.3 Herbal medicine plus UDCA and SAMe* versus *UDCA and SAMe*			
Wang et al. [36], 2016	Danyu Decoction	MD −27.30 [− 51.21, − 3.39]	
Li et al. [14], 2015	Yinchenhao Decoction	MD −19.00 [− 22.74, − 15.26]	
Su et al. [60], 2015	Lidan Decoction	MD − 14.85 [− 21.51, − 8.19]	
Zheng [65], 2019	Yinchen Decoction	MD − 18.79 [− 20.85, − 16.73]	
Mao and He [67], 2014	Qingganlidan Decoction	MD −11.20 [− 14.27, − 8.13]	
*8.2.4 Herbal medicine plus UDCA, SAMe and dexamethasone versusUDCA, SAMe and dexamethasone*			
Shan et al. [50], 2016	Huashilidan Decoction	MD-2.19 [− 6.05, 1.67]	0.27
*8.2.5 Herbal medicine plus dexamethasone* versus *dexamethasone*			
Liu et al. [38], 2015	Yinchenlidan Decoction	MD-17.48 [−24.21, − 10.75]	
Zhao [41], 2011	Qingyulidan Decoction	MD-4.65 [− 8.58, − 0.72]	
Chen and Mo [55], 2005	Self prescribed prescription	MD-6.54 [− 12.97, − 0.11]	
**9. The average gestational age of the newborn**			
9.1 Herbal medicine vs. Conventional medicine			
Zhang [31], 2006	Self prescribed prescription vs. SAMe and Reduced glutathione	MD 1.80 [1.04,2.56]	
Li et al. [14], 2015	Yinchenhao Decoction vs. UDCA and SAMe	MD − 0.50 [− 1.31,0.31]	
Huang and Liu [34], 2004	Yinchenhao Decoction vs SAMe	MD 0.70 [− 0.35, 1.75]	
9.2 Herbal medicine plus Conventional medicine versus Conventional medicine			
Li et al. [14], 2015	Yinchenhao Decoctionplus UDCA plus SAMe versus UDCA plus SAMe	MD 0.40 [− 0.37, 1.17]	
Gu et al. [66], 2014	Yinchenhao Decoction plus UDCA and SAMe versus UDCA and SAMe	MD 0.46 [− 0.29, 1.21]	
Zhu and Huang [69], 2008	Yinchenhao Decoction plus UDCA versus UDCA	MD 0.70 [− 0.35, 1.75]	
**Overall (FEM,** ***I***^***2***^ **= 0%)**		**MD 0.49 [0.01, 0.97]**	**0.05**
**10. The number of premature birth**			
10.1 Herbal medicine vs. Conventional medicine			
Yang [32], 2015	Yinzhihuang oral liquid vs UDCA	RR 0.33 [0.04, 3.03]	
Zhang et al. [35], 2006	Jiaweiyinchen Decoction vs DXM	RR 0.23 [0.03, 1.97]	
**Overall (FEM,** ***I***^***2***^ **= 0%)**		**RR 0.28 [0.06, 1.27]**	**0.10**
10.2 Herbal medicine plus Conventional medicine versus Conventional medicine			
Wang et al. [36], 2016	Yinzhihuang oral liquid plus UDCA and SAMe versus UDCA and SAMe	RR 0.27 [0.08, 0.90]	
Zhou et al. [37], 2017	Yinchailishi Decoction plus UDCA and SAMe versus UDCA and SAMe	RR 0.13 [0.02, 0.94]	
Wang X et al. [39], 2016	Danyu Decoction plus UDCA versus UDCA	RR 0.30 [0.09, 1.03]	
Yu [40], 2017	Yinzhijiangdansuan Decoction plus UDCA versus UDCA	RR 0.32 [0.04, 2.91]	
Zhao [41], 2011	Qingyulidan Decoctionplus DXM, SAMe and phenobarbital versus DXM, SAMe plus phenobarbital	RR 0.45 [0.15, 1.42]	
Shen and Tao [43], 2009	Tuihuangguyuan Decoction plus SAMe versus SAMe	RR 0.50 [0.18, 1.38]	
Wen et al. [44], 2014	Self prescribed prescription plus SAMe versus SAMe	RR 0.47 [0.22, 1.01]	
Lu [45], 2013	Yinchenzhuyedihuang Decoction plus UDCA versus UDCA	RR 0.26 [0.07, 0.99]	
Tian et al. [47], 2016	Kangdanyu Decoction plus Reduced glutathione, UDCA, SAMe and DXM versus Reduced glutathione, UDCA and SAMe	RR 1.50 [0.84, 2.68]	
Ding et al. [48], 2010	Qingganhuayu Decoction plus SAMe versus SAMe	RR 0.51 [0.32, 0.82]	
Shan et al. [50], 2016	Huashilidan Decoction plus UDCA, SAMe and dexamethasone versus UDCA, SAMe and dexamethasone	RR 0.33 [0.07, 1.57]	
Lan et al. [51], 2016	Yinchenhao Decoction plus UDCA and SAMe versus UDCA and SAMe	RR 0.42 [0.16, 1.07]	
Zhang [52], 2015	Yinchenhao Decoction plus SAMe versus SAMe	RR 0.29 [0.10, 0.79]	
Yin [53], 2015	Bushenqingli Decoction plus UDCA versus UDCA	RR 0.25 [0.06, 1.08]	
Ma [56], 2010	Self prescribed prescription plus UDCA versus UDCA	RR 0.36 [0.13, 1.03]	
Su et al. [60], 2015	Lidan Decoction plus UDCA, SAMe and Magnesium isoglycyrrhizinate versus UDCA, SAMe and Magnesium isoglycyrrhizinate	RR 0.33 [0.04, 3.06]	
Wang et al. [62], 2018	Yinzhihuang granule plus UDCA, SAMe and DXM versus UDCA, SAMe and DXM	RR 0.18 [0.04, 0.75]	
Wei [63], 2016	Self prescribed prescription plus UDCA versus UDCA	RR 0.33 [0.07, 1.52]	
Zhang [64], 2017	Lidan Decoction plus UDCA versus UDCA	RR 0.29 [0.06, 1.32]	
Zheng [65], 2019	Yinchen Decoction plus UDCA, SAMe and Phenobarbital versus UDCA, SAMe and Phenobarbital	RR 0.20 [0.01, 4.04]	
Mao and He [67], 2014	Qingganlidan Decoction plus UDCA and SAMe versus UDCA and SAMe	RR 0.23 [0.07, 0.77]	
Mao [68], 2016	Yinchenhao Decoction plus UDCA versus UDCA	RR 0.49 [0.05, 5.15]	
Liu et al. [70], 2019	Wuling pill plus SAMe versus SAMe	RR 0.43 [0.12, 1.58]	
Wang et al. [71], 2015	Yinchenhao Decoction plus SAMe versus SAMe	RR 0.60 [0.17, 2.18]	
Du and Li [72], 2014	Yiguanjian Decoction plus UDCA and DXM versus UDCA and DXM	RR 0.31 [0.12, 0.82]	
**Overall (FEM,** ***I***^***2***^ **= 7%)**		**RR 0.41 [0.34, 0.51]**	**< 0.00001**

*UDCA* Ursodesoxycholic acid, *SAMe* S-adenosylmethionine, *DXM* Dexamethasone, *VK3* Vitamin K3, *MD* Mean Difference, *RR* Risk Ratio, *FEM* Fixed effect model, *REM* Random effect model

Two studies reported changes in pruritus score of the pregnancy which was based on the standard Ribalta pruritus score [34, 35]. The analysis showed potential good adjunctive effect of herbal medicine on decreasing the pruritus scores on the basis with conventional medicine (MD -0.68, 95%CI − 0.88 to − 0.47, *I*^2^ = 0%, *P* < 0.00001, 2 trials, 120 patients). Two studies reported changes in maternal serum TBA [32, 33], since the obvious statistical heterogeneity among studies (*I*^*2*^ *=* 95%), it was impossible to combine the data (MD and 95% CI were − 4.74 μmol/L, − 6.57 to − 2.91; and 1.40 μmol/L, − 0.45 to 3.25; respectively). Two trials reported stillbirths or neonatal deaths post-treatment, and both two groups reported none [34, 35]. Three studies reported the number of fetal distress [31, 34, 35], the results showed that there was no significant difference between the herbal medicine group and conventional medicine group (RR 0.77, 95% CI 0.43 to 1.39, *I*^2^ = 0%, *P =* 0.36, 3 trials, 218 patients). Another study [32] reported the numbers of neonatal asphyxia, which also showed no difference between the herbal medicine and UDCA (RR 1.00, 95% CI 0.06 to 16.76, *P =* 1.00, 1 trial, 60 patients).

Four studies reported the number of deliveries by cesarean section [31, 33–35], the results showed that there was no difference between herbal medicine and conventional medicine (RR 0.97, 95% CI 0.80 to 1.18, *I*^2^ = 0%, *P =* 0.78, 4 trials, 338 patients). One trial reported the number of postpartum hemorrhage [35] and the results showed that there was no significant difference in this outcome between herbal medicine and conventional medicine (RR 0.47, 95% CI 0.09 to 2.36, *P =* 0.36, 1 trial, 60 patients). One trial reported changes in maternal ALT (unknown as whole blood or serum) and the results showed that there was no significant difference in this outcome between herbal medicine and conventional medicine (MD -12.80 U/L, 95% CI − 25.81 to 0.21, *P =* 0.05, 1 trial, 98 patients) [31]. Four studies reported changes in maternal serum ALT and the results showed that there was no significant difference in this outcome between herbal medicine and conventional medicine (MD 0.90, 95% CI − 5.10 to 6.90, *I*^2^ = 67%, *P =* 0.77, 4 trials, 300 patients) [32–35]. Two studies reported changes in maternal serum AST and the results showed that there was no significant difference in this outcome between herbal medicine and conventional medicine (MD 11.20 U/L, 95% CI 7.86 to 14.54, *P* < 0.00001, 1 trial, 120 patients) [33, 35]. Three studies reported the gestational age of the newborn (MD and 95% CI were 1.80 weeks, 1.04 to 2.56; − 0.50 weeks, − 1.31 to 0.31; 0.70 weeks, − 0.35 to 1.75; respectively) [31, 33, 34], meta-analysis of all the above studies could not be conducted due to the obvious statistical heterogeneity. Another trials reported the numbers of the preterm births, which showed no differences between herbal medicine and conventional medicine (RR 0.28, 95% CI 0.06 to 1.27, *I*^2^ = 0%, *P* = 0.10, 12 trials, 120 patients) [32, 35].

#### Combination of herbal medicine and conventional medicine vs. conventional medicine alone

Thrity-nine trials contributed data to this comparison [33, 36–73].

##### Pruritus score

Fifteen trials reported pruritus score of the pregnancy which was based on the standard Ribalta pruritus score [37, 38, 41–44, 46, 48, 51, 53, 59, 66, 70, 71, 73]. The post-treatment score could not be synthesized due to the obvious statistical heterogeneity. Subgroup analysis showed potential good adjunctive effect of herbal medicine on decreasing the pruritus scores on the basis with SAMe (MD -0.91, 95% CI − 1.10 to − 0.72, *I*^2^ = 80%, 7 trials, 638 women), UDCA (MD -0.58, 95% CI − 0.79 to − 0.36, *I*^2^ = 56%, 4 trials, 273 women), combination of SAMe and dexamethasone (MD -0.69, 95% CI − 0.80 to − 0.57, *I*^2^ = 0%, 2 trials, 276 women), and the combination of SAMe and UDCA (MD -1.68, 95% CI − 1.99 to − 1.37, 1 trial, 80 women), (MD -0.77, 95% CI − 1.01 to − 0.53, 1 trial, 60 women).

##### The serum TBA value

Twenty-three trials reported the serum TBA before and post-treatment [33, 36–41, 45, 46, 50–55, 57, 60, 63, 65–67, 71, 73]. The score could not be synthesised due to the obvious statistical heterogeneity. Subgroup analysis showed potential good adjunctive effect of herbal medicine on decreasing the serum TBA scores on the basis with UDCA (MD − 3.99 μmol/L, 95% CI − 4.24 to − 3.74, *I*^2^ = 0%, 7 trials, 522 women), SAMe (MD − 7.33 μmol/L, 95% CI − 8.50 to − 6.15, *I*^2^ = 0%, 4 trials, 230 women), dexamethasone (MD − 23.30 μmol/L, 95% CI − 24.61 to − 21.99, 1 trial, 86 women), combination of UDCA and SAMe (MD − 7.62 μmol/L, 95% CI − 12.97 to − 2.27, *I*^2^ = 98%, 7 trials, 652 women), and the combination of UDCA and SAMe and dexamethasone (MD − 3.54 μmol/L, 95% CI − 4.03 to − 3.05, *I*^2^ = 8%, 4 trials, 417 women).

##### The number of adverse birth events

Six trials reported no stillbirths or neonatal deaths post-treatment [53, 55, 61, 63, 69, 71]. One trial reported one neonatal deaths post-treatment in conventional medicine group [60].

Twenty-four trials reported the number of fetal distress [36–38, 40, 41, 43, 45, 47, 49–51, 53, 60, 62–65, 67–73], 18 trials reported the number of asphyxia neonatorum [37, 39–41, 43, 45, 47–53, 56, 61, 65, 67, 71], 22 trials reported the number of cesarean section [33, 36–41, 43, 45, 47, 48, 53, 55, 56, 61, 63, 64, 67, 69, 70, 72, 73], and another 14 trials reported the number of postpartum hemorrhage [39–41, 43, 47, 50, 53, 55, 60, 61, 65, 68, 70, 72]. Significantly lower incidence of fetal distress (RR 0.41, 95% CI 0.32 to 0.51, *I*^2^ = 0%, 24 trials, 1915 women), asphyxia neonatorum (RR 0.35, 95%CI 0.25 to 0.49, *I*^2^ = 0%, 18 trials, 1492 women), cesarean section (RR 0.73, 95% CI 0.63 to 0.85, *I*^2^ = 65%, 22 trials, 1974 women), postpartum hemorrhage (RR 0.45, 95% CI 0.28 to 0.72, *I*^2^ = 0%, 14 trials, 1130 women) were observed in the combination group compared to the conventional medicine group.

##### Liver function blood test results

Twenty-five trials reported the serum ALT values [33, 36, 38, 39, 41–43, 45, 46, 50, 52–55, 58–60, 63, 65–67, 69–71, 73]. The data could not be synthesized due to the obvious statistical heterogeneity. Results from single study or subgroup meta-analysis showed potential good adjunctive effect of herbal medicine on decreasing the serum ALT values on the basis of UDCA (MD -19.91 U/L, 95% CI − 35.42 to − 4.39, *I*^2^ = 99%, 9 trials, 625 women), dexamethasone (MD -15.05 U/L, 95% CI − 19.59 to − 10.51, *I*^2^ = 72%, 4 trials, 432 women), and the combination of UDCA and SAMe (MD -18.94 U/L, 95% CI − 20.91 to − 16.97, *I*^2^ = 34%, 5 trials, 506 women), the combination of UDCA, SAMe and dexamethasone (MD -8.69 U/L, 95% CI − 13.16 to − 4.22, 1 trial, 96 women). Six trials reported there is no statistical difference between the combination treatment and SAMe alone in post-treatment serum ALT value (MD -15.14 U/L, 95% CI − 30.79 to 0.50, *I*^2^ = 98%, 6 trials, 410 women). Detail of the results were shown in Table 2.

Eighteen trials reported the serum AST values [33, 36, 38, 41, 46, 50, 52, 53, 55, 56, 58, 60, 63, 65–67, 70, 71]. Results from single study or subgroup meta-analysis showed potential good adjunctive effect of herbal medicine on decreasing the serum AST values on the basis with the combination of UDCA and SAMe (MD -16.38 U/L, 95% CI − 20.49 to − 12.27, *I*^*2*^ = 79%, 5 trials, 476 women), dexamethasone (MD -9.23 U/L, 95% CI − 16.71 to − 1.76, *I*^*2*^ = 81%, 3 trials, 346 women). There is no statistical difference of herbal medicine on decreasing the serum AST values on the basis with UDCA, SAMe, and the combination of UDCA and SAMe and dexamethasone. Detail of the results were also shown in Table 2.

##### The average gestational age of the newborn

One trial reported the average gestational age of the newborn which indicated that there was no difference between thecombination group and conventional medicine group (MD 0.49 weeks, 95% CI 0.01 to 0.97, 3 trials, 263 women) [33, 66, 69].

##### The number of premature birth

Twenty-six trials reported the number of premature birth [32, 36, 37, 39–41, 43–45, 47, 48, 50–53, 60, 62–65, 67, 68, 70–72]. Significantly lower incidence of Premature birth was observed in the combination group than thecontrol group (RR 0.41, 95% CI 0.34 to 0.51, *I*^*2*^ = 7%, 26 trials, 2199 women).

### Adverse events

Five trials reported the number of adverse events [48, 53, 55, 68, 70]. One trial reported six cases of nausea, headache and flushin in treatment group [48]. One trial reported one case of loose stool, disappeared in the following course, two cases of nausea [53]. One trial reported no adverse events during the treatment [55]. One trial reported one case of headache and one case of tachycardia in treatment group, and one case of tachycardia and one case of constipation in control group [68]. Another trial reported two cases of nausea, two cases of acid reflux, one case of bloating in treatment group, and one case of acid reflux, two cases of bloating, one case of nausea, one case of rash in control group [70].

### Funnel plot

**Fig. 3:**
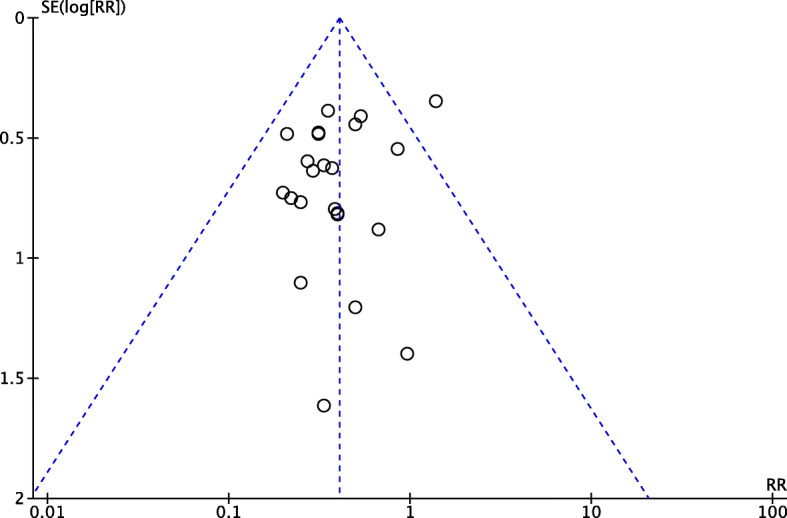
Funnel plot assessing outcomes of the number of fetal distress reported in 24 randomized controlled trials

A funnel plot analysis of 24 trials was performed to examine outcome for the serum TBA value of patients. The result showed potential asymmetry (Fig. 3). The potential asymmetry may be caused by small study effects, different methodological quality, or even Heterogeneity in intervention effects.

### Trial sequential analysis (TSA)

**Fig. 4:**
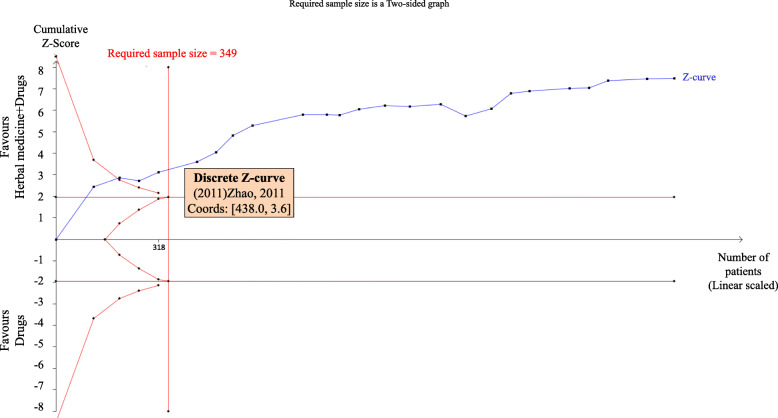
Trial Sequential Analysis results on decreasing numbers of fetal distress

We conducted TSA with the data from the two meta-analyses above. For the outcome of numbers of fetal distress in comparing herbal medicine combined conventional medicine to conventional medicine alone, TSA illustrated that the cumulative Z-curve across the traditional boundary of 5% significance (horizontal line) as well as the monitoring boundaries (inward sloping curves) (see Fig. 4). After the sixth study, the significance testing had been performed each time a new trial was added to the meta-analysis, which means the sample size achieved the required 349 participants and we had enough power to confirm the evidence (that with adjunction of herbal medicine, the therapy may decrease 12% more fetal distress) controlling for the risk of random error.

**Fig. 5:**
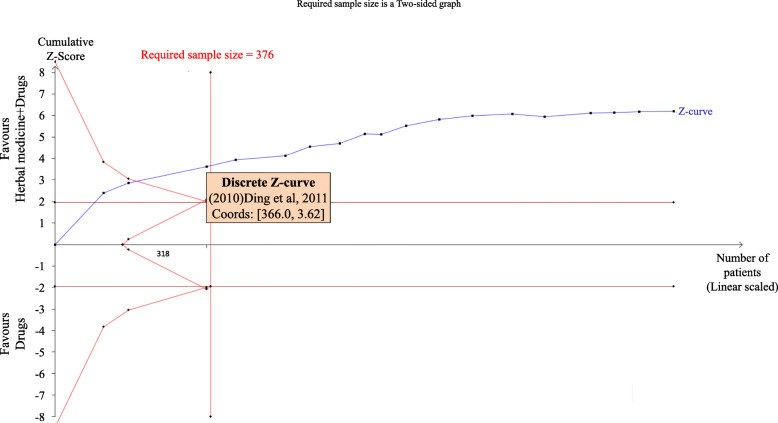
Trial Sequential Analysis results on decreasing numbers of asphyxia neonatorum

For the outcome of numbers of asphyxia neonatorum in the same comparison, the result was similar with the two crossings. TSA also illustrated that the cumulative Z-curve across the horizontal line and the inward sloping curves (see Fig. 5), which means the sample size achieved the required 376 participants and we had enough power to confirm the evidence (that the combination therapy may decrease 10% more cases of asphyxia neonatorum).

## Discussion

### Summary of main findings

Totally 43 trials with 3556 women were included, all of them were assessed as having high risk of bias. Results from these trials showed potential better effect of herbal medicine when combined with conventional medicine on decreasing pruritus scores, reducing adverse birth events (which was supported by the TSA results) and improving the liver functions according to the serum TBA, ALT, AST values compared to conventional medicine alone. However, due to the poor methodology of the included studies and the obvious statistical heterogeneity among trials, quality of the evidence for all these outcomes were “low” and “very low” according to the GRADE assessment (see Tables 3 and 4). When compared to conventional medicine, the single application of herbal medicine showed no better/worse effect for the condition, we could not draw any firm conclusion on this type of comparison due to the small sample size of involved trials. Furthermore, few data were reported to indicate the adverse events in herbal medicine group, which also made the safety of the herbal medicine was unclear.

**Table 3:** Summary of finding table of herbal medicine compared to conventional medicine for women with intrahepatic cholestasis in pregnancy

**Herbal medicine compared to conventional medicine for women with intrahepatic cholestasis in pregnancy**						
**Patient or population:** Women with intrahepatic cholestasis in pregnancy **Settings:** Outpatient department/ Inpatient department **Intervention:** Herbal medicine **Comparison:** Conventional medicine						
**Outcomes**	**Illustrative comparative risks**^**a**^ **(95% CI)**		**Relative effect** **(95% CI)**	**No of Participants** **(studies)**	**Quality of the evidence** **(GRADE)**	**Comments**
	Assumed risk	Corresponding risk				
	**Conventional medicine**	**herbal medicine**				
**Ribalta pruritus score** Scale from: 0 to 4.		The mean Ribalta pruritus score in the intervention groups was **0.68 lower** (0.88 to 0.47 lower)		120 (2 studies)	⊕⊝⊝⊝ **very low**^b,c^	
**The serum TBA value**		The mean the serum TBA value in the intervention groups was **1.67 lower** (7.69 lower to 4.35 higher)		180 (2 studies)	⊕⊝⊝⊝ **very low**^b,c,d^	
**The number of fetal distress**	**192 per 1000**	**148 per 1000** (83 to 267)	**RR 0.77** (0.43 to 1.39)	218 (3 studies)	⊕ ⊕ ⊝⊝ **low**^b^	
**The number of asphyxia neonatorum**	**33 per 1000**	**33 per 1000** (2 to 559)	**RR 1** (0.06 to 16.76)	60 (1 study)	⊕ ⊕ ⊝⊝ **low**^c,e^	

GRADE Working Group grades of evidence

**High quality:** Further research is very unlikely to change our confidence in the estimate of effect

**Moderate quality:** Further research is likely to have an important impact on our confidence in the estimate of effect and may change the estimate

**Low quality:** Further research is very likely to have an important impact on our confidence in the estimate of effect and is likely to change the estimate

**Very low quality:** We are very uncertain about the estimate

*CI* Confidence interval, *RR* Risk ratio

^a^The basis for the **assumed risk** (e.g. the median control group risk across studies) is provided in footnotes. The **corresponding risk** (and its 95% confidence interval) is based on the assumed risk in the comparison group and the **relative effect** of the intervention (and its 95% CI)

^b^There were very serious limitations of methodological quality of included trials according to the risk of bias assessment

^c^Too small sample size

^d^There were very serious statistical heterogeneity among included trials

^e^There were serious limitations of methodological quality of included trials according to the risk of bias assessment

**Table 4:** Summary of finding table of combination of herbal medicine and conventional medicine compared to conventional medicine for women with intrahepatic cholestasis in pregnancy

**Combination of herbal medicine and conventional medicine compared to conventional medicine for women with intrahepatic cholestasis in pregnancy**						
**Patient or population:** Women with intrahepatic cholestasis in pregnancy **Settings:** **Intervention:** Combination of herbal medicine and conventional medicine **Comparison:** Conventional medicine						
**Outcomes**	**Illustrative comparative risks**^**a**^ **(95% CI)**		**Relative effect** **(95% CI)**	**No of Participants** **(studies)**	**Quality of the evidence** **(GRADE)**	**Comments**
	Assumed risk	Corresponding risk				
	**Conventional medicine**	**Combination of herbal medicine and conventional medicine**				
**Ribalta pruritus score** Scale from: 0 to 4.		The mean Ribalta pruritus score in the intervention groups was **0.83 lower** (0.98 to 0.67 lower)		1327 (15 studies)	⊕⊝⊝⊝ **very low**^b,c^	
**The serum TBA value**		The mean the serum TBA value in the intervention groups was **7.62 lower** (12.97 to 2.27 lower)		652 (23 studies)	⊕⊝⊝⊝ **very low**^b,c^	
**The number of fetal distress**	**207 per 1000**	**85 per 1000** (66 to 106)	**RR 0.41** (0.32 to 0.51)	1915 (24 studies)	⊕ ⊕ ⊝⊝ **low**^b^	
**The number of asphyxia neonatorum**	**155 per 1000**	**54 per 1000** (39 to 76)	**RR 0.35** (0.25 to 0.49)	1492 (18 studies)	⊕ ⊕ ⊝⊝ **low**^b^	

GRADE Working Group grades of evidence

**High quality:** Further research is very unlikely to change our confidence in the estimate of effect

**Moderate quality:** Further research is likely to have an important impact on our confidence in the estimate of effect and may change the estimate

**Low quality:** Further research is very likely to have an important impact on our confidence in the estimate of effect and is likely to change the estimate

**Very low quality:** We are very uncertain about the estimate

*CI* Confidence interval, *RR* Risk ratio

^a^The basis for the **assumed risk** (e.g. the median control group risk across studies) is provided in footnotes. The **corresponding risk** (and its 95% confidence interval) is based on the assumed risk in the comparison group and the **relative effect** of the intervention (and its 95% CI)

^b^There were very serious limitations of methodological quality of included trials according to the risk of bias assessment

^c^There were very serious statistical heterogeneity among included trials

### Compare to the previous studies

The results of a meta-analysis preliminarily showed that traditional Chinese medicine combined with conventional medicine (or physical therapy) had a certain therapeutic effect on neonatal jaundice in treatment of recurrent spontaneous abortion [74]. Another meta-analysis showed that Yinzhihuang Oral Liquid was more effective in improving itching symptoms of pregnant women, reducing serum total bilirubin, total bilirubin, glycocholic acid and increasing neonatal weight of newborns than the control group in treatment of intrahepatic cholestasis of pregnancy [75]. The third meta-analysis showed that Yinchenhao Decoction had better effect on treating neonatal jaundice, shortening recovery time of serum total bilirubin and jaundice subsidence time than that of conventional medicine alone [76]. All these findings indicated that herbal medicine may have effect on improving the liver function of pregnancy women, which was consistent with our findings.

Since we did not find any other review focused on this disease through the literature searching, this is the probably the first study assessed the herbal medicine for ICP and the current evidence may support the effectiveness of combination of herbal medicine and conventional medicine for decreasing the maternal pruritus scores, the serum TBA, and the number of fetal distress, asphyxial events or asphyxia neonatorum events related to this condition.

### Implications for practice

Though we only got “very low” quality evidence to support the adjunctive effect of herbal medicine in treating ICP. The results showed a statistically meaningful advantage of herbal medicine on improving the symptoms or reducing adverse birth events when combined with conventional medicine. Considering the uncertain safety of the herbal product, we suggest the application of herbal medicine would be recommended in consideration of the expertise and experience of the clinician. Treatment duration would be 10 to 14 days according to this review. The most frequently used prescription in our review is Yinchenhao Decoction, in which Yinchen and Zhizi are the core component. Bian found that Yinchenhao decotion can induce liver fibrosis by dimethylnitrosamine, reduce hydroxyproline and improve liver function and hepatic histology after 2 weeks of treatment in rats [77]. Mentimes, a systematic review indicated that Yinchenhao decotion can significantly improve cholestasis by reducing elevated serum markers [78]. Practitioners may choose to use modified Yinchenhao Decoction in treating this condition.

### Implications for future studies

There are some deficiencies in this study that should be improved in future studies. In terms of scheme design and method application, most studies did not give a clear introduction to the method of randomization and blinding. Although the blind method may not be easy to operate for patients due to the difficulty in the operation of herbal medicine placebo, a blind method should be implemented for the outcome assessors or statisticians to avoid bias. Secondly, in terms of the data analysis, missing data should be handled with appropriate statistical methods. Reporting of the trial should follow the standard of CONSORT. Besides, five databases have been searched, but no studies that are published in English can be included, which suggests that relevant studies are possibly published mainly in Chinese. Since the access to databases such as Allied and Complementary Medicine (AMED) and the Cumulative Index to Nursing and Allied Health Literature (CINAHL) is not available for searching within the region of our researchers, there might be the possibility to miss studies. More English databases should be considered to be searched in the future.

High quality studies are needed to verify the adjuvant efficacy and safety of Chinese herbal medicine. Meanwhile, cost effect analysis may be considered to be conducted in the future.

## Conclusions

This review found “very low” quality evidence which support the effectiveness of combination of herbal medicine and conventional medicine for decreasing the maternal pruritus scores, the serum TBA, and the number of adverse birth events. TSA analysis showed the results of benefit of combination of herbal medicine and conventional medicine for decreasing the number of fetal distress and asphyxia neonatorum had enough statistical power. More high-quality trials are still needed to prove the superior effect and safety of herbal medicine as adjunctive treatment for this disease.

## Supplementary information

**Additional file 1.** Supplementary Material 1. The ingredients of included herbal patents.

## Data Availability

All data generated or analysed during this study are included in this published article and its supplementary information files.
