# Supplementary material for: Oral herbal medicine for women with intrahepatic cholestasis in pregnancy: a systematic review of randomized controlled trials
Source: BMC Complement Med Ther. 2020 Oct 7;20:303. doi: 10.1186/s12906-020-03097-x (PMC7542867; doi:10.1186/s12906-020-03097-x)
Supplement: Supplementary file 1 — Additional file 1. Supplementary Material 1. The ingredients of included herbal patents. [file 12906_2020_3097_MOESM1_ESM.doc]

Supplementary material 1. The ingredients of included herbal patents

| Herbal patents | Ingredient |
| --- | --- |
| Yinzhihuang oral liquid | Yinchen (*Artemisiacapillaris thumb)*, Zhizi (*Gardenia jasminoides* Ellis), Huangqin (*Scutellaria baicalensis* Georgi) and Jinyinhua (*Lonicera japonica* Thund) |
| Yinzhihuang granule | Yinchen (*Artemisiacapillaris thumb)*, Zhizi (*Gardenia jasminoides* Ellis), Huangqin (*Scutellaria baicalensis* Georgi) and Jinyinhua (*Lonicera japonica* Thund) |
| Wuling pill | Chaihu (*Bupleurum chinensis* DC), Lingzhi (*Ganoderma lucidum* (Leyss. Ex Fr.) Karst.), Danshen (*Salvia miltiorrhiza* Bge) and Wuweizi (*Schisandra chinesis* (Turcz.) Baill) |
